# Supplementary figures and images for: Identification of Recurrent Chromosome Breaks Underlying Structural Rearrangements in Mammary Cancer Cell Lines
Source: Genes (Basel). 2022 Jul 11;13(7):1228. doi: 10.3390/genes13071228 (PMC9319013; doi:10.3390/genes13071228)

A

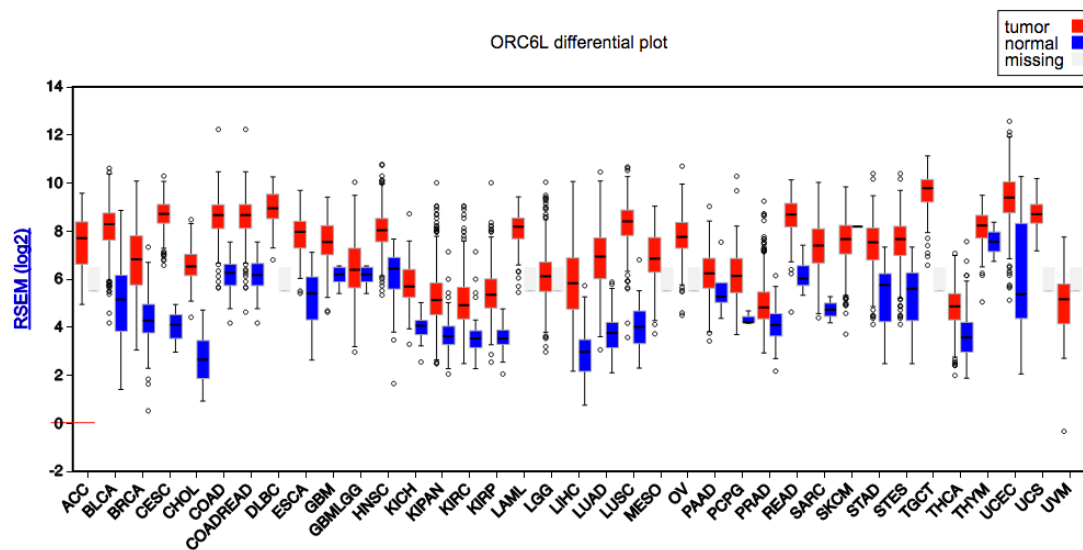

B

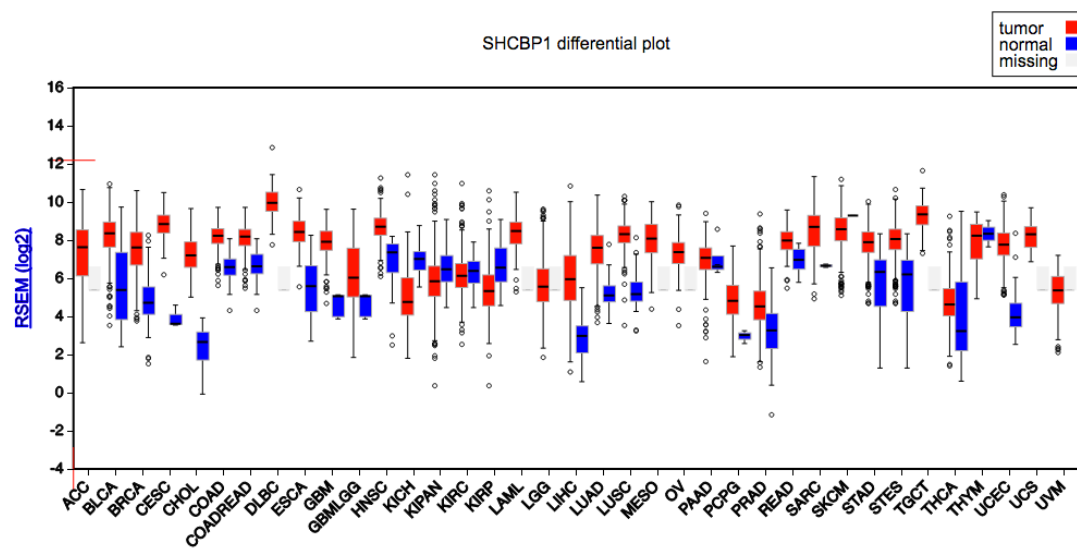

Figure S1. FIREBROWSE gene expression level across human tissues ORC6L (A) and SHCBP1 (B).

Supplement: Supplementary file 1 [file genes-13-01228-s001.zip › FigureS1.pdf]
